# The Risk of BPPV, Meniere’s Disease, and Vestibular Neuronitis in Patients with Gout: A Longitudinal Follow-Up Study Using a National Health Screening Cohort

**DOI:** 10.3390/jcm12010185

**Published:** 2022-12-26

**Authors:** Hyo Geun Choi, So Young Kim, Juyong Chung

**Affiliations:** 1 Department of Otorhinolaryngology-Head & Neck Surgery, Hallym University College of Medicine, Anyang 14068, Republic of Korea; 2 Department of Otorhinolaryngology-Head & Neck Surgery, CHA Bundang Medical Center, CHA University, Seongnam 13488, Republic of Korea; 3 Department of Otorhinolaryngology, Wonkwang University School of Medicine, 895 Muwang-ro, Iksan 54538, Republic of Korea

**Keywords:** gout, benign paroxysmal positional vertigo, Meniere’s disease, association, risk, comorbidity

## Abstract

This study evaluated the impact of pre-existing gout on the occurrence of benign paroxysmal positional vertigo (BPPV), Meniere’s disease, and vestibular neuronitis, with the goal of identifying novel associations of gout with other comorbid diseases. The 2002–2019 Korean National Health Insurance Service Health Screening Cohort data were retrospectively analyzed. 23,827 patients with gout were matched to 95,268 controls without gout for age, sex, income, region of residence, and index date. The occurrence of BPPV, Meniere’s disease, and vestibular neuronitis was evaluated in both groups. The hazard ratios (HRs) of gout for BPPV, Meniere’s disease, and vestibular neuronitis were calculated using a stratified Cox proportional hazard model. Participants with gout demonstrated a 1.13-fold higher risk of BPPV (95% CI, 1.06–1.21, *p* < 0.001) and a 1.15-fold higher risk of Meniere’s disease (95% CI, 1.15–1.37, *p* < 0.001) than the matched control group. However, the HR for vestibular neuronitis was not significantly higher in the gout group (adjusted HR = 1.06, 95% CI, 0.93–1.21, *p* = 0.391). A previous history of gout was related to a higher risk of BPPV and Meniere’s disease. Additional studies are necessary to elucidate the mechanism underlying the relationship between gout and comorbid diseases such as BPPV and Meniere’s disease.

## 1. Introduction

Gout is the most prevalent type of inflammatory arthritis in the adult population [1], and its prevalence and incidence have recently increased, becoming a growing challenge worldwide [2]. Gout results from hyperuricemia, which causes the deposition of monosodium urate crystals in and around the joints [2]. The prevalence of gout has been reported to range from <1% to 6.8%, and its incidence rate has been found to vary from 0.58 to 2.89 per 1000 person-years [2]. Men and older populations have higher prevalence and incidence rates of gout [3]. Obesity and comorbidities such as hypertension, hyperlipidemia, and kidney disease are important risk factors for gout [4]. Patients with gout have been widely established to have elevated risks for chronic kidney disease, cardiovascular disease, and acute stroke [2]. In addition, newly identified associations of gout with other comorbidities, including metabolic syndrome [5,6], erectile dysfunction [7,8,9], atrial fibrillation [10,11,12,13], obstructive sleep apnea [14,15], osteoporosis [16], and venous thromboembolism [17,18,19,20], have been reported in recent studies.

Vertigo is among the most frequent complaints encountered in clinical practice, with a reported lifetime prevalence of 7.4% in adults [21]. The most frequent peripheral causes of vertigo, in descending order, are benign paroxysmal positional vertigo (BPPV), vestibular neuronitis, and Meniere’s disease [22,23]. BPPV is the most frequent peripheral vestibular disorder in adults and has a lifetime prevalence of 2.4% [24]. Its main characteristic is short and repetitive vertigo within a minute caused by changes in head position. BPPV occurs because of free-floating otoconial debris that is dislodged and enters into the lumen of the semicircular canals [24,25]. Known risk factors of BPPV include osteoporosis, vitamin D deficiency, and old age [26]. Meniere’s disease is a cochleovestibular disorder characterized by intermittent vertigo that lasts for minutes to hours, accompanied by fluctuating sensorineural ear fullness, tinnitus, and hearing loss [27]. Meniere’s disease has an estimated prevalence of around 50 to 200 cases per 100,000 population [28]. Endolymphatic hydrops is the most common primary pathophysiology; in particular, circulatory disturbances in the inner ear can lead to abnormal endolymph homeostasis [27]. In addition, vestibular neuritis, which presents the second most frequent etiology of peripheral vertigo, is characterized by severe rotatory vertigo and spontaneous nystagmus that may last for several hours to days [29]. The incidence of vestibular neuritis is 3.5 per 100,000 population [29]. Most frequently, vestibular neuritis is caused by the reactivation of a neurotropic virus (e.g., herpes zoster virus or herpes simplex virus type 1 and 2) [29].

Some studies have reported that hyperuricemia was positively correlated with BPPV [30,31]. A recent meta-analysis reported an association between BPPV and elevated serum uric acid levels [32]. However, the literature contains limited data on the relationship of gout with the causes of peripheral vertigo. Pathological mechanisms capable of explaining the relationship of gout with peripheral vertigo have not yet been identified, but the following assumptions can be made. Higher serum uric acid levels can produce ROS, which can damage blood vessels and then interfere with the supply of blood to the inner ear. Thus, occlusion of the vertebrobasilar artery, which supplies blood to the inner ear, may cause peripheral vertigo such as Meniere’s disease, vestibular neuritis, and BBPV [33].

This study evaluated the risk of BPPV, Meniere’s disease, and vestibular neuronitis in gout patients through a nationwide population-based cross-sectional study.

## 2. Materials and Methods

### 2.1. Ethics

This study received approval from the Hallym University ethics committee (2019-10-023). The requirement for written informed consent was waived by the Institutional Review Board. All processes in this study adhered to the regulations and guidelines of the ethics committee of Hallym University.

### 2.2. Study Population and Participant Selection

Data from the Korean National Health Insurance Service-Health Screening Cohort data have been described elsewhere in the literature [34].

**Figure 1:**
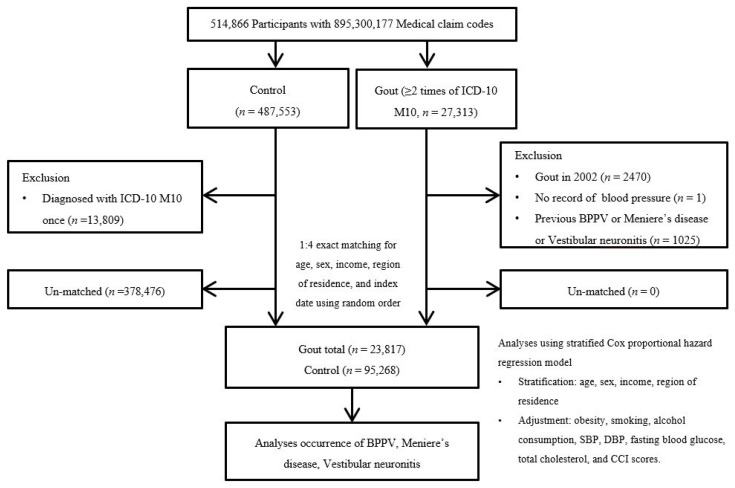
A schematic diagram of the participant selection process. From 514,866 total participants, 23,817 participants with gout were matched with 95,268 control participants according to age, sex, income, and region of residence. Abbreviation: ICD-10 = International classification of disease-10; CCI = Charlson comorbidity index; SBP, Systolic blood pressure; DBP, Diastolic blood pressure.

Participants with gout were selected from 514,866 participants with 895,300,177 medical claim codes from 2002 through 2019 (*n* = 27,313). The control group was defined as including participants who were not defined as having gout from 2002 through 2019 (*n* = 487,553). To select the participants with gout who were diagnosed for the first time, participants with gout diagnosed in 2002 were excluded (washout period, *n* = 2470). Participants with gout who had no records of blood pressure were excluded (*n* = 1). Control participants were excluded if the participants were diagnosed with the International Classification of Diseases, 10th revision code of M10 s (*n* = 13,809). Participants with gout were matched at a 1:4 ratio for age, sex, income, and region of residence. A random number order was used to sort the control participants, who were then selected from top to bottom. Therefore, we assumed that the matched control participants were evaluated at the same time as the matched participants with gout (index date); for this reason, we excluded participants in the control group who died before the index date. In both the control and gout groups, participants with a history of BPPV, Meniere’s disease, or vestibular neuronitis prior to the index date were excluded; for this reason, 1025 participants were excluded from the gout group. The matching procedure excluded 378,476 control participants. Finally, 23,817 of the participants with gout were 1:1 matched with 95,268 control participants (Figure 1).

### 2.3. Definition of Gout

Participants with gout were defined as those who visited a hospital or clinic with a gout diagnosis (ICD-10: M10) ≥2 times. This definition was adopted from an earlier study [3].

### 2.4. Definition of Benign Paroxysmal Positional Vertigo

Participants with BPPV were defined as those treated with the ICD-10 code H811 ≥2 times by a neurologist or otolaryngologist.

### 2.5. Definition of Meniere’s Disease

We defined Meniere’s disease using the ICD-10 code H810. Specifically, we included participants who were treated for Meniere’s disease ≥2 times and who underwent audiometric examinations (claim code: E6931-E6937, F6341-F6348).

### 2.6. Definition of Vestibular Neuronitis

Vestibular neuronitis was defined as treatment with the ICD-10 codes H812 ≥2 times by a neurologist or otolaryngologist.

### 2.7. Covariates

Five-year intervals were used to define age groups: 40–44…, and 85+ years old, resulting in 10 age groups. Five income groups were defined, with class 5 corresponding to the highest income and class 1 to the lowest income. Regions of residence were classified as urban (Seoul, Busan, Daegu, Incheon, Gwangju, Daejeon, and Ulsan) and rural (Gyeonggi Province, Gangwon Province, North and South Chungcheong Provinces, North and South Jeolla Provinces, North and South Gyeongsang Provinces, and Jeju Province).

Participants were classified as current smokers, past smokers, and non-smokers based on their responses regarding their current smoking status. We categorized alcohol consumption on the basis of the frequency of alcohol consumption (<1 time a week or ≥1 time a week). Obesity was measured using BMI (body mass index, kg/m^2^). BMI was categorized as <18.5 kg/m^2^ (underweight), ≥18.5 to <23 kg/m^2^ (normal), ≥23 to <25 kg/m^2^ (overweight), ≥25 to <30 kg/m^2^ (class 1 obesity), and ≥30 kg/m^2^ (class 2 obesity) based on the Asia-Pacific criteria following the Western Pacific Regional Office 2000 [35]. We also measured systolic blood pressure (mmHg), diastolic blood pressure (mmHg), fasting blood glucose (mg/dL), and total cholesterol (mg/dL).

The Charlson Comorbidity Index (CCI) has been widely used as a measurement of the disease burden based on 17 comorbidities. Each participant was given a score reflecting the number and severity of diseases. The CCI was analyzed as a continuous variable (0 (no comorbidities) through 29 (multiple comorbidities)) [36].

### 2.8. Statistical Analyses

We carried out propensity score (PS) overlap weighting to reflect the covariate balance and effective sample size. The PS was calculated by multivariable logistic regression with all covariates. To calculate overlap weighting, participants with gout were weighted by the probability of PS, and control participants were weighted by the probability of 1-PS. Overlap weighting was calculated as ranging between 0 and 1, achieving exact balance and optimizing precision. The standardized differences between before and after weighting were used to compare differences in general characteristics between the gout and control groups (Table 1).

A PS overlap-weighted Cox proportional hazard regression model was utilized to analyze the overlapping weighted hazard ratios (HRs) of gout for BPPV, Meniere’s disease, and vestibular neuronitis. In these analyses, crude (simple), and adjusted (for the CCI, total cholesterol, fasting blood glucose, systolic and diastolic blood pressure, alcohol consumption, smoking, and obesity) were generated (Table 2). The analyses were stratified by matching variables (e.g., sex, age, income, and region of residence). The log-rank test was conducted with Kaplan–Meier curves.

For the subgroup analyses using the stratified Cox-proportional hazards model, we divided participants by age (<60 vs. ≥60 years old), sex (male vs. female), obesity (underweight, normal weight, overweight, class 1 obesity, class 2 obesity), and fasting blood glucose (<100 mg/dL; ≥100 mg/dL).

In two-tailed analyses, significance was indicated by a *p*-value less than 0.05. SAS version 9.4 (SAS Institute Inc., Cary, NC, USA) was utilized for the statistical analysis.

## 3. Results

The general characteristics of gout and control participants are summarized in Table 1. The two groups showed comparable distributions in terms of age, sex, income, and region of residence region prior to weighting. Likewise, no significant differences between the gout and control groups were found for smoking status, obesity, alcohol consumption, systolic and diastolic blood pressure, fasting blood glucose, total cholesterol, and CCI scores after weighting.

In total, 3.21% (583/18,168) of participants with gout and 2.85% (518/18,168) of the control group had histories of BPPV (SD = 0.02, Table 1). The gout and control groups had no significant difference in the proportion of participants with Meniere’s disease (1.81% vs. 1.45%, SD = 0.03) or vestibular neuronitis (0.81% vs. 0.77%, SD = 0.01).

Gout showed a 1.12-fold greater hazard ratio (HR) for BPPV (95% CI, 1.04–1.22) in the crude model (*p* = 0.004) and a 1.13-fold greater HR (95% CI, 1.06–1.21) in the adjusted model (*p* < 0.001) (Table 2). A higher HR of gout for participants with BPPV was found in the <60-year-old subgroup in the crude model (HR = 1.18; 95% CI, 1.05–1.33, *p* = 0.005) and in the adjusted model (HR = 1.17; 95% CI, 1.06–1.29, *p* = 0.001). Subgroup analyses of men, women, participants with normal weight, participants who were overweight, participants who had fasting blood glucose <100 mg/dL, and participants who had fasting blood glucose ≥100 mg/dL showed a significant association of gout with BPPV in the crude and adjusted models (*p* < 0.05) (Table 2).

Gout was associated with an HR of 1.26 for Meniere’s disease in the crude model (95% CI, 1.13–1.40, *p* < 0.001) and in the adjusted model (95% CI, 1.15–1.37, *p* < 0.001) (Table 3). The higher HR of gout for Meniere’s disease was consistently found in several subgroups (Table 3).

Gout was not significantly associated with vestibular neuronitis in either the crude model (HR = 1.09; 95% CI, 0.93–1.28, *p* = 0.274) or the adjusted model (HR = 1.06; 95% CI, 0.93–1.21, *p* = 0.391) (Table 4). The subgroup analysis did not show any association of gout with vestibular neuronitis in either the crude model or the adjusted model (all *p* > 0.05) (Table 4).

**Figure 2:**
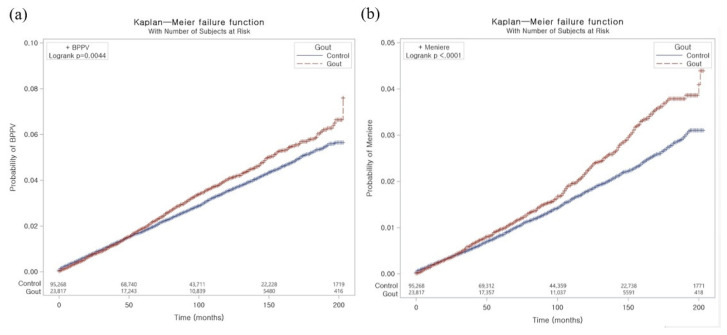
The Kaplan–Meier failure curve and cumulative incidence function for the occurrence of BPPV (**a**) and Meniere’s disease (**b**).

The Kaplan–Meier failure curve and cumulative incidence function for the occurrence of BPPV and Meniere’s disease during the 200-month period are presented in Figure 2. The rate of BPPV was significantly higher in the gout group than in the control group (*p* = 0.0044). According to the log-rank test, individuals with gout had a significantly higher cumulative incidence of Meniere’s disease than those in the comparison cohort (*p* < 0.001).

## 4. Discussion

This nationwide population-based cohort study showed higher risks of BPPV and Meniere’s disease in individuals with gout, but gout was not associated with a higher risk of vestibular neuronitis. To the authors’ best knowledge, this is the first study investigating the risk of specific types of peripheral vertigo such as BPPV, Meniere’s disease, and vestibular neuronitis in patients with gout. One study in Taiwan found that gout was positively correlated with peripheral vertigo, but in that study, peripheral vertigo did not include Meniere’s disease and no data were reported regarding the association between gout and specific vestibular disorders, such as BPPV, Meniere’s disease, and vestibular neuritis [33].

Several studies have been conducted on the association between hyperuricemia and BPPV. First, Adam reported that male African patients with BPPV showed higher serum uric acid levels [30]. This was supported by other studies in other countries [31,32,37,38,39,40,41,42,43]. A study in Turkey found that elevated serum uric acid levels were positively correlated with BPPV, with a 3.3-fold risk elevation per 1-unit increase in the serum uric acid level [31]. Their study found that a cutoff serum uric acid level of 4 mg/dL had a sensitivity of 72% and a specificity of 60% [31]. Additionally, serum uric acid levels increased during vertigo attacks but decreased significantly 1 month post-attack in BPPV patients [31]. On the contrary, two studies including European women identified similar serum uric acid levels in control and BPPV groups [43,44], and some studies in Asia stated that lower uric acid levels were found in BPPV patients [45,46]. Discrepancies in study participants’ ethnicity, gender, and/or geography could account for these differences [32]. Significantly higher serum uric acid levels were observed among BPPV patients than among controls (OR = 0.78; 95% CI, 0.15–1.41; *p* = 0.015) in a recent meta-analysis that included 12 studies [32].

The pathophysiological mechanisms underlying the relationship between gout and comorbid diseases such as BPPV and Meniere’s disease remain incompletely understood. Several studies have reported the mechanisms through which elevated serum uric acid levels contribute to the occurrence of BPPV. One proposed mechanism is that the buildup of purine crystal deposits from free-floating otoconial debris in the semicircular canals might cause BPPV in patients with gout [33]. Furthermore, higher serum uric acid levels can also cause gelatinous matrix inflammation, which has been linked to otoconia [47,48]. In addition, elevated uric acid levels induce the release of inflammatory mediators, which cause harmful reactive oxygen species (ROS) to be produced. The production of ROS can damage blood vessels and interfere with blood supply [49,50]. Thus, the relationship between gout and cardiovascular disease has been thoroughly characterized, and further evidence has been shown that gout leads to a greater risk of other vascular diseases [2]. Therefore, the occlusion of the vertebrobasilar arteries supplying the inner ear may cause peripheral vertigo, and this hypothesis can serve as a potential explanation for the association of gout with peripheral vertigo (e.g., BPPV and Meniere’s disease) [33]. In Meniere’s disease, histopathological temporal bone studies have shown that circulatory disturbances might cause abnormal endolymph homeostasis. [51,52]. A greater volume of the endolymphatic fluid causes the cochlear lateral wall, including the capillary network, to be compressed against the bony labyrinth, and ischemic reperfusion damage can be caused by this increase in cochlear outflow resistance. Therefore, individuals with existing problems of microvascular structures caused by gout might be more vulnerable to inner ear damage due to endolymphatic hydrops [53]. In particular, thiazide diuretics, which are widely used to treat Meniere’s disease, can also affect the occurrence of gout. A meta-analysis of cohort studies confirmed that hypertension and diuretic therapy predispose individuals to the development of gout [54]. The present study also showed an increased risk of Meniere’s disease in gout patients. Therefore, future studies will need to examine the bidirectional association of gout with Meniere’s disease. Vestibular neuronitis is thought to be associated with viral infection. In this study, there was no association between gout and vestibular neuronitis. Serum uric acid levels, which are associated with oxidative stress and antioxidant status, were lower in patients with various neurological disorders, including multiple sclerosis [55]. Serum uric acid levels were clearly reduced in patients with viral central nervous system infection but restored with effective treatment [55]. Therefore, in patients with vestibular neuronitis caused by the reactivation of a neurotropic virus, serum uric acid levels are more likely to have decreased than to be high.

From a healthcare perspective, our paper presents a valuable discovery that BPPV and Meniere’s disease show a novel association with gout. Gout is a worldwide challenge, with increasing prevalence and incidence [2]. In Korea, the incidence and prevalence of gout increased from 2007 to 2015 [3]. The prevalence of gout increased from 3.49 per 1000 persons in 2007 to 7.58 per 1000 persons in 2015. The incidence was 1.52 in 2009 and rose to 1.94 per 1000 persons in 2015 [3]. Higher uric acid levels, rising age, alcohol consumption, obesity, hypertension, and diuretic use are known to be correlated with the risk of gout [3]. The increasing prevalence and incidence of obesity and comorbidities are likely to make a major contribution to the burden imposed by gout [2]. The link between gout and comorbidities is complex. Some comorbid diseases predispose to gout and others can occur as a result of gout [2]. A large study from the UK investigated the temporal relationships between the occurrence of comorbid diseases and a first gout diagnosis [4]. Renal disease, hyperlipidemia, and hypertension were identified as risk factors for gout, and gout increased the risk of subsequent cardiovascular disease and renal disease. The bidirectional relationship between gout and chronic kidney disease was confirmed by cohort studies from the UK, USA, and Canada [2]. Gout patients in the UK were at a more than 50% risk of being diagnosed with peripheral vascular disease during the 10 years before and after a gout diagnosis [4]. Thus, peripheral vascular disease has a bidirectional relationship with gout. As vascular pathology in the inner ear may cause peripheral vertigo, such as BPPV and Meniere’s disease, these disorders can also increase the risk of gout due to vascular pathology. Additionally, the risk of BPPV and Meniere’s disease increased over time after a gout diagnosis and was greater in people with a normal BMI than in those who were overweight or obese, suggesting that gout exacerbates the risk of BPPV and Meniere’s disease independent of BMI (Table 2 and Table 3). This pattern appeared not only in BMI, but also in age, sex, and blood glucose level. The risk of BPPV and Meniere’s disease was greater in individuals less than 60 years of age than in those over 60 years old. Thus, gout increases the risk of BPPV and Meniere’s disease independently of age, sex, and blood glucose level. Although the bidirectional relationship between gout and peripheral vestibular diseases such as BPPV and Meniere’s disease has not yet been elucidated, vascular pathology, which can cause these diseases, can be a predisposing factor for gout. Therefore, the discovery of novel gout-associated comorbidities and efforts to reduce the risk of these comorbidities are also essential for the management of gout. Moreover, efforts to reduce blood uric acid levels can contribute to preventing the occurrence or recurrence of comorbid diseases such as BPPV and Meniere’s disease, for which individuals with gout have an increased risk.

In the present study, men had an almost 4-fold higher incidence of gout than women (79% vs. 20%) (Table 1). The reason for male predominance remains unclear, although a possible explanation is that estrogen’s uricosuric effect can reduce the risk of gout in premenopausal women [56]. Postmenopausal women showed a relative risk of 1.26 for gout, with premenopausal women as the reference group [57]. In addition, differences in men’s diet and lifestyle patterns can partially explain the higher incidence and prevalence of gout in men.

This study had several strengths. We used a large study population and took steps to minimize bias. This population-based cohort study provided data on temporal relationships between the occurrence of BPPV, Meniere’s disease, and vestibular neuronitis after the first diagnosis of gout. A long-term longitudinal study was conducted from 2002 to 2019, and matching and adjustment were carried out to control a number of potential confounding factors. Furthermore, demographic factors, socioeconomic factors, lifestyle factors, and comorbidities were considered. The statistical analysis of the incidence of BPPV and MD according to the timeline suggests important health implications regarding an increasing risk over time for these novel comorbidities of gout.

Our study has several limitations. It was not possible to evaluate vestibular function tests or the degree of hearing loss because this information was not available in the claims database. Moreover, the etiologic factors were heterogeneous for gout and peripheral vestibular disorders such as BPPV, Meniere’s disease, and vestibular neuronitis. Additionally, it was not possible to analyze disease severity and duration or treatment options. Although many variables were adjusted, other confounders that were not analyzed in this study, such as stress, nutritional status, and physical activity, may have affected the results. Furthermore, this study was conducted only in Korean cohorts. Koreans are ethnically homogeneous. Differences related to ethnicity in terms of diet, comorbid disease patterns, and genetics are able to increase the susceptibility to gout [2]. In this study, there is a limitation in that these ethnic-related factors were not considered. Therefore, future research should seek to validate our results in populations of more diverse races. Last, the pathophysiological mechanisms underlying the relationship between gout and comorbid diseases such as BPPV and Meniere’s disease are not fully understood, and further research will therefore be needed in the future.

## 5. Conclusions

In conclusion, this study showed that gout was associated with an increased risk of BPPV and Meniere’s disease after adjusting for confounding factors. Our study is meaningful in revealing that BPPV and Meniere’s disease are novel comorbidities associated with gout. Thus, controlling gout will also contribute to lowering the risk of comorbid diseases such as BPPV and Meniere’s disease.

## Figures and Tables

**Table 1:** General Characteristics of Participants.

Characteristics	Before Overlap Weighting Adjustment			After Overlap Weighting Adjustment		
	Gout	Control	Standardized Difference	Gout	Control	Standardized Difference
Age (*n*, %)			0.00			0.00
40–44	591 (2.48)	2364 (2.48)		444 (2.44)	444 (2.44)	
45–49	2072 (8.70)	8288 (8.70)		1547 (8.51)	1547 (8.51)	
50–54	3602 (15.12)	14,408 (15.12)		2719 (14.97)	2719 (14.97)	
55–59	4688 (19.68)	18,752 (19.68)		3582 (19.72)	3582 (19.72)	
60–64	4080 (17.13)	16,320 (17.13)		3131 (17.23)	3131 (17.23)	
65–69	3382 (14.20)	13,528 (14.20)		2589 (14.25)	2589 (14.25)	
70–74	2645 (11.11)	10,580 (11.11)		2027 (11.16)	2027 (11.16)	
75–79	1687 (7.08)	6748 (7.08)		1300 (7.15)	1300 (7.15)	
80–84	815 (3.42)	3260 (3.42)		632 (3.48)	632 (3.48)	
85+	255 (1.07)	1020 (1.07)		198 (1.09)	198 (1.09)	
Sex (*n*, %)			0.00			0.00
Male	18,948 (79.56)	75,792 (79.56)		14,372 (79.11)	14,372 (79.11)	
Female	4869 (20.44)	19,476 (20.44)		3796 (20.89)	3796 (20.89)	
Income (*n*, %)			0.00			0.00
1 (lowest)	3456 (14.51)	13,824 (14.51)		2637 (14.51)	2637 (14.51)	
2	2944 (12.36)	11,776 (12.36)		2252 (12.40)	2252 (12.40)	
3	3609 (15.15)	14,436 (15.15)		2756 (15.17)	2756 (15.17)	
4	5038 (21.15)	20,152 (21.15)		3841 (21.14)	3841 (21.14)	
5 (highest)	8770 (36.82)	35,080 (36.82)		6681 (36.78)	6681 (36.78)	
Region of residence (*n*, %)			0.00			0.00
Urban	10,062 (42.25)	40,248 (42.25)		7668 (42.21)	7668 (42.21)	
Rural	13,755 (57.75)	55,020 (57.75)		10,499 (57.79)	10,499 (57.79)	
Obesity † (*n*, %)			0.28			0.00
Underweight	317 (1.33)	2415 (2.53)		269 (1.48)	269 (1.48)	
Normal	5969 (25.06)	33,032 (34.67)		4901 (26.98)	4901 (26.98)	
Overweight	6511 (27.34)	26,813 (28.14)		5057 (27.84)	5057 (27.84)	
Obese I	9960 (41.82)	30,612 (32.13)		7231 (39.80)	7231 (39.80)	
Obese II	1060 (4.45)	2396 (2.52)		709 (3.90)	709 (3.90)	
Smoking status (*n*, %)			0.08			0.00
Nonsmoker	12,449 (53.79)	49,257 (53.23)		9771 (53.78)	9771 (53.78)	
Past smoker	5483 (23.69)	19,833 (21.43)		4218 (23.22)	4218 (23.22)	
Current smoker	5212 (22.52)	23,447 (25.34)		4178 (23.00)	4178 (23.00)	
Alcohol consumption (*n*, %)			0.09			0.00
<1 time a week	12,085 (50.74)	52,581 (55.19)		9380 (51.63)	9380 (51.63)	
≥1 time a week	11,732 (49.26)	42,687 (44.81)		8787 (48.37)	8787 (48.37)	
Systolic blood pressure (Mean, SD)	129.56 (16.85)	127.37 (16.29)	0.13	129.04 (14.81)	129.04 (7.32)	0.00
Diastolic blood pressure (Mean, SD)	80.16 (11.03)	78.83 (10.61)	0.12	79.83 (9.69)	79.83 (4.77)	0.00
Fasting blood glucose (Mean, SD)	102.56 (28.28)	103.03 (30.88)	0.02	102.68 (25.60)	102.68 (12.51)	0.00
Total cholesterol (Mean, SD)	198.40 (40.52)	195.26 (37.85)	0.08	197.55 (35.71)	197.55 (17.06)	0.00
CCI score (Mean, SD)	1.20 (1.84)	0.99 (1.71)	0.12	1.15 (1.58)	1.15 (0.83)	0.00
BPPV (*n*, %)	762 (3.20)	2715 (2.85)	0.02	583 (3.21)	518 (2.85)	0.02
Meniere’s disease (*n*, %)	434 (1.82)	1382 (1.45)	0.03	329 (1.81)	264 (1.45)	0.03
Vestibular neuronitis (*n*, %)	195 (0.82)	714 (0.75)	0.01	148 (0.81)	139 (0.77)	0.01

Abbreviations: SD, standard deviation; CCI, Charlson Comorbidity Index; BPPV, benign paroxysmal positional vertigo; † Obesity (BMI, body mass index, kg/m^2^) was categorized as <18.5 (underweight), ≥18.5 to <23 (normal), ≥23 to <25 (overweight), ≥25 to <30 (obese I), and ≥30 (obese II).

**Table 2:** Crude and adjusted hazard ratios of Gout for BPPV with subgroups according to age and sex with subgroups according to age, sex, obesity, and fasting blood glucose.

Independent Variables	IR Per 1000 Person-Year	IRD Per 1000 Person-Years (95% Confidence Interval)	Hazard Ratios for BPPV (95% Confidence Interval)			
			Crude †	*p*-Value	Adjusted †‡	*p*-Value
Total participants (*n* = 119,085)						
Gout	4.27	0.47 (0.15 to 0.80)	1.12 (1.04–1.22)	0.004 *	1.13 (1.06–1.21)	<0.001 *
Control	3.79		1		1	
Age < 60 (*n* = 54,765)						
Gout	3.51	0.55 (0.17 to 0.93)	1.18 (1.05–1.33)	0.005 *	1.17 (1.06–1.29)	0.001 *
Control	2.96		1		1	
Age ≥ 60 (*n* = 64,320)						
Gout	5.29	0.37 (−0.19 to 0.93)	1.07 (0.96–1.20)	0.208	1.09 (0.99–1.19)	0.070
Control	4.92		1		1	
Men (*n* = 94,740)						
Gout	3.61	0.29 (−0.05 to 0.62)	1.09 (0.99–1.20)	0.095	1.08 (1–1.17)	0.049 *
Control	3.32		1		1	
Women (*n* = 24,345)						
Gout	7.07	1.32 (0.40 to 2.24)	1.23 (1.06–1.42)	0.005 *	1.23 (1.09–1.38)	<0.001 *
Control	5.76		1		1	
Underweight (*n* = 2732)						
Gout	6.21	2.33 (−0.71 to 5.37)	1.58 (0.85–2.94)	0.147	1.29 (0.82–2.03)	0.269
Control	3.88		1		1	
Normal weight (*n* = 29,706)						
Gout	4.84	0.43 (−0.55 to 1.42)	1.32 (1.14–1.54)	<0.001 *	1.28 (1.14–1.44)	<0.001 *
Control	3.65		1		1	
Overweight (*n* = 39,001)						
Gout	4.32	0.46 (−0.16 to 1.08)	1.12 (0.96–1.30)	0.149	1.13 (1–1.28)	0.049 *
Control	3.86		1		1	
Obese I (*n* = 40,572)						
Gout	3.95	0.08 (−0.43 to 0.59)	1.02 (0.90–1.16)	0.740	1.04 (0.93–1.16)	0.521
Control	3.87		1		1	
Obese II (*n* = 3456)						
Gout	3.34	−0.60 (−2.27 to 1.06)	0.84 (0.53–1.33)	0.466	0.86 (0.56–1.32)	0.489
Control	3.95		1		1	
Fasting blood glucose < 100 mg/dL (*n* = 68,732)						
Gout	4.34	0.51 (0.09 to 0.92)	1.13 (1.02–1.25)	0.018 *	1.13 (1.04–1.23)	0.004 *
Control	3.84		1		1	
Fasting blood glucose ≥ 100 mg/dL (*n*= 50,353)						
Gout	4.16	0.43 (−0.08 to 0.94)	1.11 (0.98–1.27)	0.099	1.12 (1.01–1.25)	0.033 *
Control	3.73		1		1	

Abbreviation: IR, incidence rate; IRD, incidence rate difference; * Stratified cox proportional hazard regression model, Significance at *p* < 0.05; † Models were stratified by age, sex, income, and region of residence. ‡ The model was adjusted for obesity, smoking, alcohol consumption, systolic blood pressure, diastolic blood pressure, fasting blood glucose, total cholesterol, and CCI scores.

**Table 3:** Crude and adjusted hazard ratios of Gout for Meniere’s disease with subgroups according to age and sex with subgroups according to age, sex, obesity, and fasting blood glucose.

Independent Variables	IR Per 1000 Person-Year	IRD Per 1000 Person-Years (95% Confidence Interval)	Hazard ratios for Meniere’s Disease (95% Confidence Interval)			
			Crude †	*p*-Value	Adjusted †‡	*p*-Value
Total participants (*n* = 119,085)						
Gout	2.41	0.53 (0.27 to 0.80)	1.26 (1.13–1.40)	<0.001 *	1.26 (1.15–1.37)	<0.001 *
Control	1.91		1		1	
Age < 60 (*n* = 54,765)						
Gout	1.90	0.44 (0.03 to 0.86)	1.39 (1.18–1.64)	<0.001 *	1.37 (1.2–1.58)	<0.001 *
Control	1.37		1		1	
Age ≥ 60 (*n* = 64,320)						
Gout	3.08	0.37 (−0.19 to 0.93)	1.17 (1.01–1.35)	0.035 *	1.17 (1.03–1.32)	0.012 *
Control	2.64		1		1	
Men (*n* = 94,740)						
Gout	1.92	0.23 (−0.01 to 0.46)	1.13 (0.99–1.29)	0.065	1.11 (1–1.24)	0.06
Control	1.70		1		1	
Women (*n* = 24,345)						
Gout	4.45	1.66 (1.00 to 2.32)	1.59 (1.32–1.92)	<0.001 *	1.61 (1.37–1.90)	<0.001 *
Control	2.79		1		1	
Underweight (*n* = 2732)						
Gout	4.07	2.18 (0.01 to 4.34)	2.14 (0.98–4.67)	0.056	1.83 (0.98–3.40)	0.058
Control	1.89		1		1	
Normal weight (*n* = 29,706)						
Gout	2.75	0.80 (0.34 to 1.26)	1.41 (1.16–1.73)	<0.001 *	1.32 (1.13–1.54)	<0.001 *
Control	1.95		1		1	
Overweight (*n* = 39,001)						
Gout	2.55	0.63 (0.19 to 1.08)	1.33 (1.09–1.63)	0.005 *	1.32 (1.11–1.56)	0.002 *
Control	1.91		1		1	
Obese I (*n* = 40,572)						
Gout	2.16	0.29 (−0.07 to 0.64)	1.15 (0.97–1.38)	0.117	1.18 (1.01–1.38)	0.043 *
Control	1.88		1		1	
Obese II (*n* = 3456)						
Gout	1.59	−0.24 (−1.38 to 0.89)	0.87 (0.45–1.70)	0.693	0.97 (0.53–1.78)	0.922
Control	1.83		1		1	
Fasting blood glucose < 100 mg/dL (*n* = 68,732)						
Gout	2.57	0.72 (0.42 to 1.01)	1.39 (1.21–1.59)	<0.001 *	1.39 (1.24–1.56)	<0.001 *
Control	1.85		1		1	
Fasting blood glucose ≥ 100 mg/dL (*n* = 50,353)						
Gout	2.16	0.15 (−0.22 to 0.52)	1.08 (0.90–1.29)	0.418	1.06 (0.91–1.23)	0.467
Control	2.01		1		1	

Abbreviation: IR, incidence rate; IRD, incidence rate difference; * Stratified cox proportional hazard regression model, Significance at *p* < 0.05; † Models were stratified by age, sex, income, and region of residence. ‡ The model was adjusted for obesity, smoking, alcohol consumption, systolic blood pressure, diastolic blood pressure, fasting blood glucose, total cholesterol, and CCI scores.

**Table 4:** Crude and adjusted hazard ratios of Gout for Vestibular neuronitis with subgroups according to age and sex with subgroups according to age, sex, obesity, and fasting blood glucose.

Independent Variables	IR Per 1000 Person-Year	IRD Per 1000 Person-Years (95% Confidence Interval)	Hazard Ratios for Vestibular Neuronitis (95% Confidence Interval)			
			Crude †	*p*-Value	Adjusted †‡	*p*-Value
Total participants (*n* = 119,085)						
Gout	1.07	0.09 (−0.07 to 0.25)	1.09 (0.93–1.28)	0.274	1.06 (0.93–1.21)	0.391
Control	0.98		1		1	
Age < 60 (*n* = 54,765)						
Gout	0.94	0.13 (−0.06 to 0.33)	1.16 (0.93–1.46)	0.187	1.11 (0.92–1.34)	0.277
Control	0.81		1		1	
Age ≥ 60 (*n* = 64,320)						
Gout	1.25	0.04 (−0.24 to 0.31)	1.03 (0.82–1.29)	0.805	1.01 (0.84–1.20)	0.952
Control	1.22		1		1	
Men (*n* = 94,740)						
Gout	0.98	0.04 (−0.14 to 0.22)	1.04 (0.87–1.26)	0.645	1.01 (0.87–1.17)	0.944
Control	0.94		1		1	
Women (*n* = 24,345)						
Gout	1.48	0.30 (−0.11 to 0.71)	1.26 (0.92–1.72)	0.148	1.26 (0.97–1.65)	0.08
Control	1.17		1		1	
Underweight (*n* = 2732)						
Gout	1.51	0.32 (−1.31 to 1.95)	1.28 (0.38–4.33)	0.690	0.98 (0.41–2.34)	0.972
Control	1.19		1		1	
Normal weight (*n* = 29,706)						
Gout	0.95	0.03 (−0.28 to 0.34)	1.03 (0.74–1.43)	0.866	1.06 (0.83–1.34)	0.656
Control	0.92		1		1	
Overweight (*n* = 39,001)						
Gout	0.98	0.07 (−0.23 to 0.37)	1.07 (0.78–1.47)	0.663	1.03 (0.79–1.33)	0.838
Control	0.91		1		1	
Obese I (*n* = 40,572)						
Gout	1.18	0.13 (−0.14 to 0.39)	1.12 (0.88–1.43)	0.342	1.12 (0.91–1.38)	0.299
Control	1.05		1		1	
Obese II (*n* = 3456)						
Gout	1.19	−0.35 (−1.38 to 0.67)	0.77 (0.36–1.64)	0.503	0.79 (0.4–1.55)	0.489
Control	1.54		1		1	
Fasting blood glucose < 100 mg/dL (*n* = 68,732)						
Gout	1.05	0.04 (−0.17 to 0.25)	1.04 (0.85–1.28)	0.682	1.02 (0.86–1.20)	0.815
Control	1.00		1		1	
Fasting blood glucose ≥ 100 mg/dL (*n* = 50,353)						
Gout	1.12	0.17 (−0.09 to 0.42)	1.17 (0.91–1.51)	0.208	1.11 (0.9–1.36)	0.336
Control	0.95		1		1	

Abbreviation: IR, incidence rate; IRD, incidence rate difference; † Models were stratified by age, sex, income, and region of residence. ‡ The model was adjusted for obesity, smoking, alcohol consumption, systolic blood pressure, diastolic blood pressure, fasting blood glucose, total cholesterol, and CCI scores.

## Data Availability

Releasing of the data by the researcher is not legally permitted. All data are available from the database of the Korea Center for Disease Control and Prevention. The Korea Center for Disease Control and Prevention allows data access, at a particular cost, for any researcher who promises to follow the research ethics. The data of this article can be downloaded from the website after agreeing to follow the research ethics.
